# Neutrophil extracellular traps in rheumatoid arthritis: pathogenic mechanisms and therapeutic potential

**DOI:** 10.3389/fimmu.2025.1717671

**Published:** 2025-11-25

**Authors:** Cheng-Liang Mao, Chen-Huan Yu, Song-Lin Jiang, Zhen-Hua Ying, Xing-Yi Zhu

**Affiliations:** 1 Key Laboratory for Green Pharmaceutical Technologies and Related Equipment of Ministry of Education, Zhejiang Key Laboratory of Green Manufacturing Technology for Chemical Drugs, College of Pharmaceutical Sciences, Zhejiang University of Technology, Hangzhou, China; 2 Zhejiang Provincial Key Laboratory of Traditional Chinese Medicine Cultivation for Arthritis Diagnosis and Treatment, Zhejiang Provincial People’s Hospital, Hangzhou Medical College, Hangzhou, China; 3 Hangzhou Institute of Medicine, Chinese Academy of Sciences, Hangzhou, China; 4 Key Laboratory of Experimental Animal and Safety Evaluation, Hangzhou Medical College, Hangzhou, China

**Keywords:** rheumatoid arthritis, neutrophil extracellular traps, autoimmune disease, anti-citrullinated protein antigens, inflammatory cytokines

## Abstract

Rheumatoid arthritis (RA) is a chronic autoimmune disease marked by synovial inflammation, joint destruction, and systemic complications, eventually leading to a high rate of disability, but its exact pathogenesis remains unclear. Neutrophil extracellular traps (NETs) are chromatin fibers released by activated neutrophils during infection/inflammation, containing histones, antimicrobial proteins, and granule components. Under physiological conditions, NETs trap pathogens and act as a pivotal anti-infective mechanism of the innate immune response. During the development of RA, NET components act as danger-associated molecular patterns (DAMPs) to activate NLRP3 inflammasomes and the complements in effector lymphocytes, amplifying inflammation; NETs promote the RA-related autoantibody production in B cells, such as anti-citrullinated protein antibodies (ACPAs) and rheumatoid factor (RF), fueling autoimmunity, while ACPAs further induce NETosis, creating a vicious feedback loop; NETs facilitate the release of pro-inflammatory cytokines (e.g., IL-6, IL-1β, TNF-α), exacerbating joint damage; finally, NETs activate T cells, dendritic cells, and macrophages via boosting RAGE/TLR9 pathway, thereby driving the proliferation and migration fibroblast-like synoviocytes. Notably, inhibiting NET formation (e.g., FcαRI antibody, celastrol), blocking NET-mediated inflammation (e.g., RAGE/TLR9 antagonists), and clearing NET remnants to break the pathogenic cycle (e.g., PAD enzyme inhibitors, DNase I and CD19 CAR-T trials) provide novel strategies for RA treatment. This article highlights the pathogenic role of NETs in RA, and emphasizes the potential as clinical biomarkers and therapeutic targets for RA progression. It will open avenues for novel treatments targeting NETosis or its downstream effects, potentially improving outcomes for RA and other inflammatory arthritides.

## 1 Introduction

Rheumatoid arthritis (RA) is a chronic, systemic autoimmune disorder characterized by persistent synovial inflammation, progressive joint destruction, and a wide range of extra-articular manifestations, including cardiovascular and pulmonary complications. Despite significant advancements in treatment strategies, RA remains a leading cause of disability worldwide, affecting approximately 1% of the global population (1). The pathogenesis of RA involves a complex interplay of genetic predisposition (e.g., HLA-DRB1 shared epitope alleles), environmental triggers (e.g., smoking, infections), and dysregulated immune responses (2–4). Central to RA development is the loss of immune tolerance, leading to autoantibody production, such as anti-citrullinated protein antibodies (ACPAs) and rheumatoid factor (RF), which contributes to chronic inflammation and tissue damage (5).

Neutrophils, the most abundant innate immune cells in RA synovial fluid, play a crucial role in disease initiation and progression (6). Beyond their classical functions—phagocytosis, reactive oxygen species (ROS) production, and protease secretion—neutrophils contribute to autoimmunity through the release of neutrophil extracellular traps (NETs). NETs are web-like structures composed of decondensed chromatin decorated with histones, antimicrobial peptides (e.g., LL-37, defensins), and granule proteins (e.g., myeloperoxidase, neutrophil elastase). While NETs are essential for trapping and killing pathogens during infections, their dysregulated formation in autoimmune diseases like RA exacerbates inflammation and tissue injury (7). Especially, NETs act as a major source of citrullinated autoantigens, which drive ACPA production and fuel a self-perpetuating cycle of autoimmunity in RA. Additionally, NET components function as danger-associated molecular patterns (DAMPs), activating pattern recognition receptors (e.g., TLR9, RAGE) on immune cells, thereby promoting inflammasome activation (e.g., NLRP3) and pro-inflammatory cytokine release (e.g., IL-1β, IL-6, TNF-α) (8–11). Furthermore, NETs enhance fibroblast-like synoviocyte (FLS) proliferation and osteoclastogenesis, accelerating joint erosion (9, 12). Given their multifaceted roles in RA pathogenesis, NETs have emerged as promising biomarkers and therapeutic targets. This review explores the mechanisms by which NETs contribute to RA pathogenesis, their potential as diagnostic and prognostic biomarkers, and novel therapeutic approaches targeting NETosis. Understanding these pathways may pave the way for precision medicine in RA and other autoimmune diseases characterized by aberrant NET formation.

## 2 The formation of NETs (NETosis) and their categories

The process by which neutrophils form NETs is called NETosis, which was initially considered a distinct form of cell death different from senescence, apoptosis, necrosis, and others. It has been well known that ROS mediate the formation of NETs, which are generated through both nicotinamide adenine dinucleotide phosphate (NADPH) oxidase (NOX)-dependent and NOX-independent pathways. Thus, the formation of NETs can be divided into two categories: suicidal NETs and non-suicidal NETs. Their characteristics are presented as shown in **Table 1**.

**Table 1:** The characteristics of different types of NET formation (summarized based on references (13–18) and related literature).

Categories	Suicidal NETs	Non-suicidal NETs	
		Vital NETs	Mitochondrial NETs
Activator and receptor	PMA, cholesterol and auto Abs through NOX2	*S. aureus* through TLR2 and C5a receptors *E. coli* and platelets through TLR4	GM-CSF pre-treatment and LPS/C5a stimulation
Onset after exposure	4 h	10 min	Hours to days (context-dependent)
NADPH dependent	Yes	No	No
ROS production	Yes	No	Yes
DNA origin	Nucleus	Nucleus	Mitochondria
Place of histone citrullination and chromatin decondensation	Nucleus	Vesicles	Mitochondria
Type of chromatin release	Nuclear membrane disruption	Vesicle membrane disruption	Mitochondrial membrane disruption
Antibacterial activity	Reduced	Preserved	Preserved

Suicidal NETs, also known as NADPH oxidase-dependent NETs, are the most common type and typically occur hours after neutrophils are stimulated (13). In the NOX-dependent pathway, neutrophils are activated by various stimuli, such as microorganisms, platelets, immune complexes (ICs), autoantibodies, cytokines, potent protein kinase C (PKC) activator phorbol myristate acetate (PMA), and calcium ions (Ca²^+^), which can activate neutrophils primarily through Fcγ receptors, leading to NOX-dependent ROS production, sustained PAD4 activation, and suicidal NETosis (13). The membrane-bound NOX2 in neutrophils then produces ROS, which act on azurophilic granules (AG), leading to the translocation of myeloperoxidase (MPO) and neutrophil elastase (NE) from AG to the nucleus. These enzymes promote histone degradation and chromatin decondensation in the nucleus, followed by neutrophil membrane disruption and NET formation (14). In suicidal NETosis, PAD4 activation is downstream of ROS production via NOX2, and its activity is essential for nuclear chromatin unfolding. Their hallmarks are the disruption of the nuclear and plasma membranes, leading to the release of nuclear chromatin into the cytoplasm, where it mixes with granular proteins before being expelled into the extracellular space. This process involves the activation of the Raf-MEK-ERK pathway, which triggers downstream NADPH oxidase to produce ROS and upregulates anti-apoptotic proteins, ultimately resulting in cell death (15).

Non-suicidal NETs are further divided into vital NETs and mitochondrial NETs. Among them, vital NETs occur within minutes after neutrophil stimulation and can be induced by *Staphylococcus aureus* through Toll-like receptor 2 (TLR2) ligands (16) and complement receptors, *Escherichia coli* and platelets through TLR4 (17). Regarding activation by different antigens, microbial components such as LPS (via TLR4) or lipoteichoic acid (via TLR2) potently induce vital NETosis through rapid, ROS-dependent but NOX-independent pathways, often involving spleen tyrosine kinase (Syk) and calcium signaling. This pathway often involves secondary ROS amplification via MPO and NE, culminating in dramatic chromatin expansion and cell rupture. Its characteristic lies in the extrusion of NETs via vesicles, with the plasma membrane remaining intact and cellular functions such as chemotaxis, phagocytosis, and antibacterial activity preserved. PAD4 may be activated through calcium influx independently of NOX-derived ROS, allowing for rapid NET extrusion without immediate cell lysis. This form is commonly observed in conditions such as thrombosis, systemic lupus erythematosus, RA, and autoimmune vasculitis.

Mitochondrial NETs rely on the production of ROS after granulocyte-macrophage colony-stimulating factor (GM-CSF) pre-treatment and LPS/C5a stimulation, in which the extruded DNA scaffold originates from mitochondrial DNA rather than nuclear DNA (18). This type of NETosis is distinct from suicidal NETs as it does not involve nuclear membrane disruption and maintains neutrophil viability in the early stages. It is worth noting that the granular protein components in NETs are not uniform, and different formation mechanisms can lead to the release of distinct protein groups. Mitochondrial NETosis also involves PAD4, which may contribute to the citrullination of mitochondrial proteins, although the mechanisms are less fully elucidated.

It is important to note that in the dynamic and chaotic environment of a diseased joint (or other inflammatory site), a combination of different NETotic forms is happening simultaneously, contributing collectively to the net pathological outcome. The key is not to identify one single form, but to understand the overall burden of NETs and the imbalance between their formation and clearance, which is central to diseases like RA.

## 3 NETs as drivers of RA pathogenesis

### 3.1 NETs promote the autoantigen production

NETs are the primary source of citrullinated proteins and other autoantigens in RA. In RA patients, autoantigens can emerge during the subclinical stage, with citrullinated histones being a major antigen in RA (19, 20). During the formation of NETs by neutrophils to eliminate pathogens, histone citrullination is a critical step. Citrullination is a post-translational modification process in which arginine residues in proteins are converted into citrulline by PADs. PADs are a family of enzymes that mediate protein citrullination and consist of five isozymes (PAD1–4 and PAD6). Neutrophils predominantly express PAD2 and PAD4. Under normal conditions, PAD4 remains inactive, but during oxidative stress or autoimmune responses, Ca²^+^ influx into the cell activates PAD4, leading to the citrullination of histones, NE, azurocidin, and myeloid differentiation antigens. Additionally, perforin and the complement membrane attack complex (MAC) can form pores in neutrophils, increasing intracellular Ca²^+^ levels and activating PAD4, resulting in widespread citrullination of neutrophil proteins (21). When excessive citrullination exceeds the body’s immune tolerance threshold, ACPAs are generated, triggering autoimmunity. RA patients exhibit a correlation between NETs and ACPA levels in peripheral blood (21). When ACPAs are cleared from plasma, anti-NET antibody activity also decreases (22). Conversely, ACPAs can stimulate neutrophils to release more NETs, perpetuating inflammation and creating a vicious cycle (21, 23).

**Figure 1:**
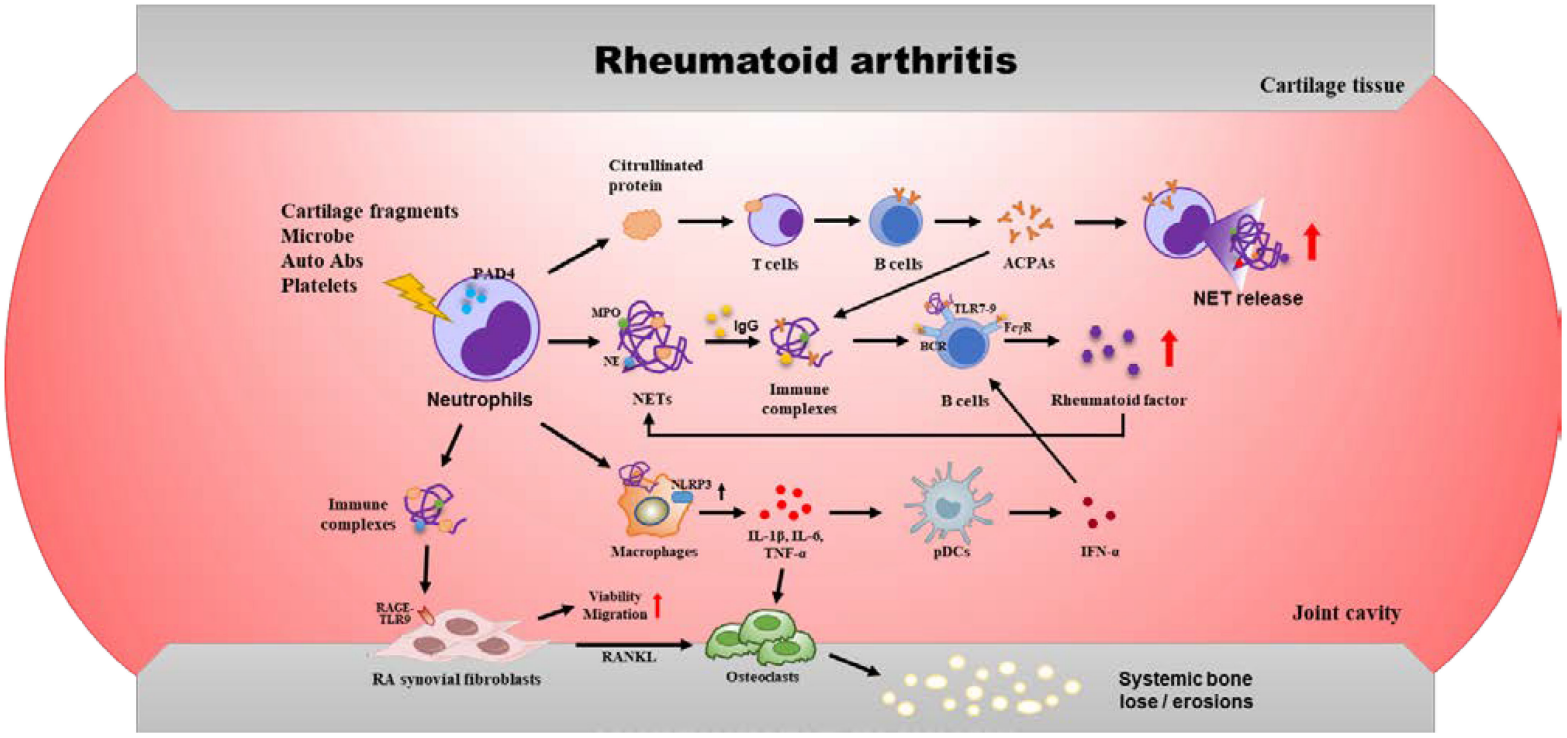
Cellular and molecular events involved in the pathogenesis of rheumatoid arthritis. NETs promote the autoantigen production, inflammatory response, and synovial fibroblast activation.

Furthermore, NETs release proteins that undergo non-enzymatic carbamylation in the inflammatory microenvironment, forming another class of autoantigens (carbamylated proteins, CarP). These promote pathogenic adaptive immunity, leading to the production of anti-CarP antibodies and anti-carbamylated NET protein antibodies, which activate macrophages to release pro-inflammatory cytokines (24, 25). This self-sustaining loop of NET formation, autoantigen release, and autoantibody production drives chronic inflammation and joint destruction in RA (**Figure 1**). Targeting NETs or their modified proteins may offer novel therapeutic strategies for RA.

### 3.2 Immune complexes containing NET components stimulate RF generation

In RA, autoantibodies, such as ACPAs, bind to citrullinated proteins within NETs, forming ICs. These ICs may also contain DNA, histones, and other NET-associated proteins, which stimulate RF production through multiple mechanisms. (1) B-Cell receptor (BCR) engagement: When RF^+^ B cells encounter IgG-containing immune complexes (e.g., ACPA-IgG bound to NETs), their BCRs recognize the Fc portion of IgG, leading to B-cell activation (26). (2) Toll-Like Receptor (TLR) stimulation: NET components (e.g., DNA, and histones) can engage TLR9 and TLR7/8 in B cells and dendritic cells, amplifying B-cell activation, and autoantibody production (27, 28). (3) Fcγ receptor (FcγR) cross-Linking: ICs can engage FcγRs on antigen-presenting cells (APCs), promoting cytokine release (e.g., BAFF, IL-6) that further drives B-cell proliferation and RF secretion (28). Conversely, RF (IgM or IgG autoantibodies against the Fc region of IgG) enhances immune complex formation, leading to complement (C3a, C5a) activation and inflammation as well as FcγR-mediated phagocyte activation, perpetuating synovial damage (14, 29). In short, there is a self-sustaining loop where NETs fuel autoantibody production, and RF amplifies immune complex deposition in joints.

### 3.3 NETs promote inflammatory response and angiogenesis

The interaction between NETs and aggregated immune cells (especially, macrophages) can initiate the synthesis of the IL-1β precursor, which is then catalyzed into mature IL-1β by the activated NLRP3 inflammasome (8). Additionally, NETs activate macrophages to release TNF-α, IL-6, and IFN-α, which stimulate plasmacytoid dendritic cells (pDCs) to produce a significant amount of IFN-α. IFN can then promote the differentiation and maturation of dendritic cells, activate T cells, and stimulate B cells to produce autoantibodies, further enhancing NET formation. NETs can promote IL-17 production by lymphocytes, particularly Th17 and γδ T cells, via interactions between NET components (e.g., histones or DNA) and pattern-recognition receptors (e.g., TLR2/4/9) (11). The DNA at the lesion site (including the DNA from NETs themselves) and the complexes formed by granular proteins in NETs can stimulate plasmacytoid dendritic cells in the synovial fluid, triggering a robust type I interferon response (30). Due to persistent inflammatory reactions and hypoxic environmental stimulation, RA is often accompanied by the angiogenesis in the synovial tissues, which accelerates the progression of synovial hyperplasia (31, 32). Specifically, the components in NETs, such as IL-8, MPO, NE and cathepsin G, cleave and release extracellular matrix-bound VEGF, while histones act as DAMPs to activate TLR signaling in endothelial cells, thereby promoting angiogenesis in cancers, atherosclerosis, myocardial infarction and wound healing. Additionally, the scaffold of extracellular DNA facilitates the localized concentration and delivery of these pro-angiogenic factors (33). Although the role of NETs in promoting angiogenesis has been widely confirmed in various diseases such as tumors and cardiovascular ischemia-reperfusion injury (34–36), their roles in the development and progression of RA still requires further *in vitro* and *in vivo* studies for validation.

### 3.4 NETs promote synovial fibroblast activation

FLSs are the primary cells that promote synovial hyperplasia in joints. They can attract inflammatory cells to act on the synovium, exacerbating joint inflammation and tissue damage. The citrullinated proteins in NETs can serve as autoantigens to activate immune cells in the synovium, stimulating FLSs or macrophages to express and release pro-inflammatory cytokines, such as IL-8, IL-6, and IL-33. These inflammatory cytokines and various antibodies can, in turn, induce the production of NETs, leading to a loss of immune tolerance and promoting the progression of RA (12, 37).

RA-FLSs internalize NETs via the RAGE-TLR9 pathway, where NET components such as HMGB1 and DNA synergistically activate this process. Following internalization, FLSs process NET-derived antigens—including citrullinated peptides—and present them via major histocompatibility complex class II (MHC-II) to activate antigen-specific T cells, thereby promoting adaptive immune responses (11). Concurrently, NET uptake enhances pro-inflammatory cytokine and chemokine production by FLSs. Studies have shown that NETs promote the proliferation of RA-FLSs *in vitro* and significantly upregulate the expression of CTGF mRNA in RA-FLSs (12).

### 3.5 Interactions between NETs and immune cells

NETs engage in extensive crosstalk with multiple immune cells, forming a pro-inflammatory network that drives RA pathogenesis. Specifically, NET-derived components—such as citrullinated histones and cell-free DNA—act as DAMPs that activate antigen-presenting cells via Toll-like receptors (TLR2, TLR4, and TLR9), promoting the secretion of IL-1β, IL-6, and IL-23 (11). These cytokines, particularly IL-1β and IL-23, are critical for the differentiation of naïve CD4^+^ T cells into the Th17 phenotype. In turn, Th17-derived IL-17A potently stimulates neutrophil activation and NETosis, creating a self-amplifying, positive-feedback loop that exacerbates autoimmunity and chronic inflammation. These cytokines facilitate the differentiation of naïve CD4^+^ T cells into Th17 cells. In turn, Th17-derived IL-17A potently stimulates neutrophil activation and NETosis, creating a self-amplifying loop that exacerbates autoimmunity. Furthermore, NETs serve as a source of citrullinated autoantigens that are taken up by B cells and dendritic cells, leading to ACPA production and T cell priming, thereby bridging innate and adaptive immunity (27, 38). This intricate crosstalk, especially the Th17/NET axis, represents a critical pathogenic circuit in RA, the disruption of which holds significant therapeutic promise.

### 3.6 NETs activate the complement system

In addition to the mechanisms mentioned above, NETs exacerbate RA inflammation by activating the complement system. (1) PAD2 catalyzes the citrullination of type II collagen and aggrecan, leading to structural destabilization of the chondrocyte extracellular matrix and increasing its susceptibility to degradation. Meanwhile, citrullinated proteins act as DAMPs, activating the TLR4/NF-κB pathway and inducing the production of inflammatory cytokines (e.g., IL-1β, TNF-α), which promotes chondrocyte apoptosis (39). (2) During NETosis, the mtDNA released by neutrophils has pro-inflammatory properties, adding complexity to NET-mediated autoimmunity. Since mtDNA contains remnants of bacterial nucleic acids and exhibits abnormal methylation, the body may mount an immune response against mtDNA, enhancing TLR signaling and amplifying inflammatory signals, thereby triggering RA (40). (3) When NETs bind to related antibodies, they can activate the complement system via the classical pathway, further intensifying inflammatory responses (38, 41). Also, NETs (especially DNA-histone complexes) activate complement through the lectin pathway or bypass pathway.

## 4 NETs as biomarkers in RA

Given the central role of NETs in RA pathogenesis described above, their components and formation signatures have garnered significant interest as potential biomarkers for diagnosis, prognosis, and monitoring treatment response.

### 4.1 Differences in NETs formation between ACPA^+^ and ACPA^-^ patients

ACPAs are highly specific serological markers for RA, and their presence or absence defines distinct clinical subsets of the disease with different pathological features (19, 20). The formation of NETs differs significantly between ACPA^+^ and ACPA^-^ patients, primarily due to distinct autoimmune microenvironments and stimulation pathways (**Table 2**).

**Table 2:** Differences in NETosis between ACPA^+^ and ACPA^-^ patients.

Features	ACPA^+^ patients	ACPA^-^ patients
Primary Stimuli	ACPA immune complexes, FcγR activation (21)	Cytokines, microbial components, TLR signaling
NETosis Type	Predominantly suicidal NETosis (21)	vital NETosis with mitochondrial DNA
PAD4 involvement	Essential, extensive histone citrullination (21)	Less prominent, alternative modifications
ROS Source	NOX2-dependent ROS production (21)	NOX-independent and mitochondrial ROS
Genetic influence	Strong HLA association, PAD4 polymorphisms	Different genetic risk loci (e.g., TLR genes)
Clinical Correlation	More severe erosion, systemic manifestations (19, 42, 43)	Different disease activity patterns

In ACPA-Positive patients, their RA is characterized by a more severe disease course and stronger genetic association with HLA-DRB1 shared epitope alleles. ACPA immune complexes can directly activate neutrophils through Fcγ receptors (FcγRIIa and FcγRIIIa), leading to increased NOX-dependent ROS production and subsequently causing suicidal NETosis (21). The citrullinated antigens present in NETs may further amplify ACPA production, creating a self-perpetuating loop of autoimmunity (21, 23). Moreover, those patients show elevated levels of PAD4 in neutrophils, leading to extensive histone citrullination (particularly H3Arg8,17,26), which facilitates chromatin decondensation and NET release. This PAD4 activation appears to be driven by both genetic factors and the inflammatory microenvironment. Therefore, excessive NET formation in ACPA-positive patients correlates with more severe joint erosion, systemic inflammation, and extra-articular manifestations (42, 43). NETs can be served as a source of citrullinated autoantigens that can activate adaptive immune responses and perpetuate synovitis.

By contrast, ACPA-negative RA represents a more heterogeneous entity with different genetic and environmental risk factors. NET formation in these patients is primarily triggered by conventional inflammatory stimuli such as cytokines (IL-8, TNF-α), complement activation, or microbial components rather than autoantibody-mediated activation. These patients show a higher tendency for vital NETosis, where neutrophils release mitochondrial DNA-based NETs while maintaining cellular viability and functions. This process involves TLR4 and TLR8 signaling rather than Fc receptor activation. NET formation in ACPA-negative patients is less dependent on PAD4-mediated histone citrullination and more reliant on ROS production through alternative pathways, including mitochondrial ROS generation. Although NETs still contribute to inflammation, the autoantigens displayed are less likely to be citrullinated and may involve other post-translationally modified proteins (e.g., carbamylated or acetylated proteins).

Therefore, the presence of ACPA defines a distinct pathogenetic subset of RA where NET formation is driven by autoantibody-mediated neutrophil activation through Fcγ receptors, resulting in PAD4-dependent histone citrullination and suicidal NETosis. This creates a self-sustaining loop of autoantigen exposure and autoimmune amplification. In contrast, ACPA-negative patients exhibit different NETosis patterns triggered primarily by innate immune stimuli, with less emphasis on citrullination-dependent pathways. These differences underscore the importance of stratified approaches to targeting NETosis in RA therapeutics.

### 4.2 Neutrophil-derived DNA and histone components in RA progression

Neutrophil-derived DNA and histone components play a critical role in the progression of RA and demonstrate significant potential as prognostic biomarkers. During NET formation, released cell-free DNA (cfDNA) and citrullinated histones (such as H3Cit) function as DAMPs (42–44). These molecules activate TLR9 and TLR4 signaling pathways, promoting the activation of immune cells including dendritic cells and macrophages. This leads to sustained production of pro-inflammatory cytokines such as tumor necrosis factor-α (TNF-α) and interleukin-1β (IL-1β), exacerbating synovitis and joint damage. Furthermore, NET components—particularly citrullinated histones—serve as an important source of autoantigens that drive the production of anti-citrullinated protein antibodies (ACPAs), creating a self-amplifying cycle of autoimmunity that accelerates disease progression (21).

Clinically, the levels of NET-derived biomarkers in serum or synovial fluid are strongly correlated with disease activity and severity. Elevated levels of MPO-DNA complexes, cfDNA, and citrullinated histones H3/H4 (CitH3/CitH4) are frequently observed in ACPA-positive patients and are associated with more severe radiographic progression and extra-articular manifestations (42, 43). Recent studies indicate that patients with high baseline NET markers often respond poorly to conventional DMARDs, while a decrease in these markers is observed following effective biologic therapy, suggesting their utility as dynamic indicators of treatment response (42). Additionally, NET components show promise in distinguishing clinical subtypes of RA and predicting disease flares (42). Therefore, neutrophil-derived DNA and histone components contribute not only to the inflammatory and autoimmune mechanisms underlying RA but also hold considerable potential as prognostic biomarkers. Standardization of detection methods and validation of their sensitivity and specificity through large-scale longitudinal studies could enable the use of these markers for risk stratification, treatment optimization, and the development of novel NET-targeting therapies in RA.

### 4.3 Serum NETs and deoxyribonuclease I

Emerging clinical trials have revealed that abundant NETs are present in the synovial tissue, rheumatoid nodules, and skin of RA patients, which exhibit heightened sensitivity to stimuli that activate neutrophils (23, 42). The percentage of NETs in peripheral blood shows a significant positive correlation with serum levels of erythrocyte sedimentation rate (ESR), C-reactive protein (CRP), ACPAs, and IL-17. However, NETs can also be detected in large quantities in various autoimmune diseases, atherosclerosis, and tumors, they lack sufficient specificity. Therefore, NETs should be evaluated in combination with other RA-related biomarkers to improve the reliability of diagnostic results.

During the development of RA, impaired clearance of NETs is often observed. DNase I, an acidic glycoprotein with endonuclease activity primarily found in the blood and gastrointestinal tract, is a key degradation factor for NETs. DNase I may function by inhibiting the production of ROS, thereby reducing NET formation and promoting the degradation of existing NETs, though the exact mechanism remains unclear. It was found that DNase I in the synovial fluid of RA patients was nearly inactive, and serum DNase I activity was significantly reduced, showing a negative correlation with inflammatory markers such as ESR, CRP, and neutrophil count. The reduction in DNase I activity observed in RA patients results from a complex interplay of factors including molecular inhibitors (G-actin, autoantibodies), an adverse synovial environment (altered ionic composition, increased ROS, low pH, proteases), and potentially genetic regulation (hypermethylation of DNASE1 promoter region). Moreover, impaired DNase I activity leads to ineffective clearance of NETs, increasing the exposure of citrullinated autoantigens and promoting the production of ACPAs, which further exacerbates joint inflammation (44).

Although elevated levels of NETs have been observed across multiple inflammatory and autoimmune diseases—such as systemic lupus erythematosus, vasculitis, and sepsis—their utility as a specific biomarker for RA remains challenging due to limited disease specificity. Therefore, the integration of multi-component biomarkers can enhance the diagnostic and prognostic accuracy of NETs-related measurements in RA. One particularly promising combination includes serum CitH3, anti-PAD4 antibodies, MPO-DNA complexes, and DNase I activity, whose panel captures different aspects of NETosis biology. Although no study to date has comprehensively evaluated this specific combination, this panel warrants further clinical validation.

## 5 Therapeutic strategies targeting NETosis

During chronic infections and inflammatory reactions, NETs contribute to the pathogenesis of RA via diverse mechanisms. Therapeutic strategies aimed at inhibiting NET formation, clearing NET remnants and neutralizing NET-mediated inflammation to reduce aberrant NET accumulation hold promise for mitigating RA (**Table 3**).

**Table 3:** Current clinical status of targeting NETosis agents for RA treatment.

Agent	Clinical status for RA treatment	Therapeutic strategy	Target	Effects	References
Cl-amidine	Investigation	Inhibiting NET formation	PAD4	Reduce the production of citrullinated proteins	(45)
Anti-CD32a Abs, antiCD64 Abs	Investigation	Inhibiting NET formation	FcαRI, FcγRI (CD64)	Inhibit neutrophil activation and NET formation	(46, 47)
N-acetylcysteine	Investigation	Inhibiting NET formation	GSS	Reduce oxidative stress and histone citrullination in NETs	(48)
Celastrol, glycyrrhizic acid	Approved	Inhibiting NET formation	ROS	Inhibit neutrophil activation and NET formation	(49, 50)
Natural phenolics (tetrandrine, andrographolide, emodin, and quercetin)	Investigation	Inhibiting NET formation	ROS/NOX2	Inhibit neutrophil activation and NET formation	(41, 51–54)
Apigenin, tanshinone IIA	Investigation	Neutralizing NET-Mediated Inflammation	TLR9	Modulate RA-FLS activation via RAGE/TLR9-related pathways	(55, 56)
Hydroxychloroquine	Approved	Neutralizing NET-Mediated Inflammation	TLR9	Inhibit the viability and migration of RA-FLSs and inactivate pDC and B cells	(57–59)
TNF-α inhibitors (etanercept, adalimumab)	Approved	Neutralizing NET-Mediated Inflammation	TNF-α	Antagonizing TNF-α disrupts the inflammatory cascade in RA	(60–62)
IL-6R antagonists (tocilizumab)	Approved	Neutralizing NET-Mediated Inflammation	IL-6R	IL-6/IL-6R blockade attenuates IL-6-driven inflammatory signaling	(63)
T-cell co-stimulation modulators (abatacept)	Approved	Neutralizing NET-Mediated Inflammation	CTLA4	Inhibit T-cell activation	(64)
DNase I	Investigation	Clearing NET remnants	cfDNA	Reduce cfDNA in joint	(65, 66)
CD19 CAR-T	Investigation	Clearing NET remnants	B cells	Inhibit B-cell activation and antagonize TNF-α/IL-6	(67, 68)

### 5.1 Inhibiting NET formation

In the pathogenesis of RA, PADs are released into the extracellular space by activated neutrophils in the joints, triggering pro-inflammatory Th1 and Th17 responses, resulting in the persistent inflammation (21, 69). Inhibiting PAD activity can impede the formation of NETs, thereby alleviating inflammatory responses in RA, making it a novel therapeutic target for RA treatment. The PAD inhibitor Cl-amidine decreased citrullinated proteins and IL-6 in joints and serum of RA mice and mitigated the joint damage (45). Its clinical significance lies in potentially preventing the initiation and propagation of autoimmunity in high-risk or early RA patients. Moreover, cotreatment with anti-CD64 (FcγRI) antibody and PAD2 inhibitor rather than anti-CD32a (FcγRIIA) antibody and PAD4 inhibitor curbed ACPA-induced RA-FLS activation and osteoclastogenesis in RA patients (46). Additionally, FcαRI-blocking antibodies broke the cycle of NETosis and autoantibody production in IgA IC-exposed neutrophils, indicating the potential of FcαRI blockade for treating RA (47).

ROS is the major culprit of neutrophil activation. Treatment with ROS scavengers N-acetylcysteine (48) and natural phenolic compounds, such as celastrol, glycyrrhizic acid, tetrandrine, andrographolide, emodin, and quercetin (41, 49–54), counteracted oxidative stress by inactivating mTOR, MAPK, ERK, or NF-κB pathway, thereby suppressing histone citrullination in NETs and NET formation in collagen-induced rheumatoid arthritis mice. While less specific, they may benefit patients with prominent oxidative stress-driven pathology.

### 5.2 Neutralizing NET-mediated inflammation

NETs containing citrullinated peptides could be internalized by RA-FLSs through activating RAGE-TLR9 pathway, leading to the exacerbation of synovial inflammation and joint tissue damage (11). This internalization process could be modulated by apigenin and tanshinone IIA via regulating pathways involving RAGE/TLR9 to relieve RA symptoms (55, 56). Hydroxychloroquine is one of the immune-suppressants commonly used in clinical for RA treatment. It not only inhibited the proliferation, migration, and invasion of RA-FLSs but also inactivated pDC and memory B cells by suppressing TLR9 receptor (57–59). However, these findings remain at the preclinical pharmacological research stage, and their clinical efficacy still requires further evaluation. Additionally, biologics such as TNF-α inhibitors (e.g., etanercept, adalimumab, infliximab, golimumab), IL-6R antagonists (tocilizumab), and T-cell co-stimulation modulators (abatacept) effectively suppress inflammation and joint damage in RA, but such treatments tend to palliate the symptoms rather than the root causes and may increase long-term risks of infections and cardiovascular events (60–64).

### 5.3 Clearing NET remnants

Excessive cfDNA derived from NETs in serum and synovium is one of the momentous pathogenic factors for RA development. Clearing cfDNA using DNase I may be an effective strategy for the treatment of patients with impaired NET clearance or high levels of NET remnants. However, this approach faces challenges such as systemic toxicity and low cfDNA clearance efficiency. Emerging study revealed that intravenous administration of endogenous DNase I alone did not effectively alleviate joint inflammation in CIA mice (65). By conjugating biomimetic cell membranes with dendritic cationic peptides, DNase I-loaded nanogels can be targeted to the joints of mice and subsequently alleviated RA symptoms in CIA mice via suppressing the TLR9 pathway (66).

Chimeric antigen receptor T cells (CAR-T) are T cells genetically engineered to acquire the ability to target specific antigens. In 2021, CAR-T cells for the first time were successfully treated a patient with relapsed and refractory systemic lupus erythematosus, ushering in a new era in the treatment of autoimmune diseases (67). Recently, a novel autologous fourth-generation CD19 CAR-T therapy has been reported to B cells that contribute to the NET-promoting environment. This therapy not only exhibits precise CD19 targeting but also simultaneously secretes antibodies against IL-6 and TNF-α, thereby more effectively preventing refractory RA (68). Although current CAR-T therapy for RA remains in the exploratory clinical stage and requires longer-term follow-up observations to evaluate its safety, cost, target selection, it demonstrates the promising efficacy and application prospects of CAR-T cell therapy in RA, particularly in refractory cases.

### 5.4 Therapeutic implications of NETs–immune cell interactions

Targeting the interplay between NETs and immune cells offers promising therapeutic avenues for RA. Inhibition of NET formation through PAD4 inhibitors or disruption of NET structures via DNase I treatment can reduce autoantigen exposure and mitigate downstream immune activation. Additionally, biologics targeting IL-17A (e.g., secukinumab) or Th17-polarizing cytokines (e.g., IL-23 inhibitors) may break the cyclic interaction between NETs and Th17 cells. Specifically, disrupting the Th17/NET positive-feedback loop by targeting IL-17 or IL-23 could potentially halt the chronic propagation of inflammation and autoimmunity in a subset of patients. Combining NET-targeting strategies with conventional DMARDs or B cell-depleting therapies could synergistically suppress inflammation and ameliorate RA progression. Furthermore, blocking TLR signaling (e.g., TLR4 or TLR9 antagonists) can interrupt NET-induced activation of dendritic cells and macrophages, thereby dampening subsequent T and B cell responses. A critical evaluation of these strategies must consider the redundancy in immune pathways and the potential for such interventions to be most effective in patients with evidence of strong NET-immune cell engagement.

### 5.5 Toward a stratified treatment model: targeting NETosis in ‘NET-high’ RA endotypes

The heterogeneity of RA necessitates a move beyond a one-size-fits-all treatment approach. Emerging evidence suggests that patients with a high burden of NET formation, termed “NET-high” RA, may constitute a distinct pathogenetic endotype characterized by specific clinical and serological features. The combination of csDMARDs with NETosis inhibitors represents a promising therapeutic strategy for RA, particularly in patients with evidence of NET-driven pathology. This approach simultaneously targets adaptive immune activation and innate inflammatory pathways, leveraging the complementary mechanisms of csDMARDs—which modulate T-cell and B-cell responses—and NETosis inhibitors that reduce neutrophil-mediated tissue damage and autoantigen exposure. A stratified treatment model for “NET-high” RA could be guided by biomarkers such as elevated levels of serum CitH3, MPO-DNA complexes, anti-PAD4 antibodies, and/or impaired DNase I activity. By mitigating NET-related osteoclastogenesis, cartilage degradation, and autoantigen presentation, the combination therapy may not only overcome csDMARD resistance but also more effectively inhibit radiographic progression than monotherapies.

To translate this synergistic strategy into clinical practice, key research priorities must be addressed, including the development of biomarker-driven criteria (e.g., high CitH3 or MPO-DNA complexes) to identify “NET-high” RA patients, optimization of treatment timing and dosing sequences, and the creation of non-invasive NET imaging tools for real-time monitoring. However, challenges such as potential infection risks due to prolonged NETosis suppression, regulatory hurdles in demonstrating additive benefits, and the complexity of integrating these therapies with biologics in refractory RA require careful consideration through stratified clinical trials and long-term safety studies.

## 6 Conclusion and future perspectives

NETs play an intricate role in RA pathogenesis by driving autoimmunity, inflammation, and joint damage. Targeting NETs offers a promising therapeutic strategy, with several agents in preclinical and clinical development, and presents significant challenges. A major hurdle is balancing NET inhibition with host defense, as NETs are vital in combating infections. Systemic suppression of NETosis (e.g., via PAD4 inhibitors or DNase I) may increase susceptibility to pathogens, necessitating selective approaches that disrupt pathogenic NETs without compromising antimicrobial immunity. Therefore, strategies targeting specific NET components (e.g., citrullinated histones or LL-37) rather than global NET formation might mitigate this risk. Furthermore, the compositional differences between NETs generated in response to infections versus those in sterile autoimmunity like RA (e.g., variations in the profile of citrullinated proteins, antimicrobial peptides, and associated DAMPs) need to be elucidated, as this could allow for the development of highly selective therapies that disrupt pathogenic NETs without compromising antimicrobial defense (11, 21). The conceptual framework of “NET-high” endotypes provides a rationale for stratified medicine, allowing for the targeted application of NET-directed therapies in the patients most likely to benefit, thereby maximizing efficacy while minimizing systemic exposure and potential off-target effects, including compromised host defense. Exploring combination therapies (e.g., NET inhibition and conventional anti-rheumatic drugs) could also contribute to good long-term outcome. Ultimately, overcoming these challenges requires a dual strategy: (1) refining NET-targeted therapies to preserve immune function, and (2) integrating novel technologies for precision intervention. Such advances could revolutionize RA management while minimizing off-target effects.

Emerging technologies are advancing NET-focused RA research and therapy. NET imaging techniques, such as using fluorescently labeled antibodies against citrullinated histones (e.g., H3Cit) or MPO-DNA complexes, as well as the development of specific PET tracers, could enable real-time visualization of NET deposition in joints, improving disease monitoring and assessment of treatment efficacy (42, 43). Additionally, machine learning is being applied to identify NET-associated biomarkers for early RA diagnosis. Other emerging therapies, including CAR-T cell therapy, nanoparticle-based deliver system and CRISPR-based gene editing, have demonstrated encouraging clinical efficacy in early trails, numerous challenges pertaining to their safety, long-term effectiveness, and targeting precision remain to be thoroughly addressed. Consequently, their translation into routine clinical practice still requires further extensive investigation and validation. Future research must prioritize the definitive validation of NET-related biomarkers, the development of selective NETosis inhibitors that spare antimicrobial functions, and the conduct of clinical trials specifically enriched for “NET-high” patient populations. In-depth research on the factors regulating NET formation and exploring the mechanism of NET remnants in RA may provide new targets for the precise treatment and diagnosis of RA and other inflammatory arthritides, holding significant potential for clinical application.
